# Wearable near-infrared spectroscopy: reliability and sensitivity among different endurance cycling exercise intensities

**DOI:** 10.1590/1414-431X2024e13102

**Published:** 2024-03-04

**Authors:** J.S. Hasegawa, R.A. Azevedo, A.C. Silveira, A.E. Lima-Silva, R. Bertuzzi

**Affiliations:** 1 Grupo de Estudos em Aptidão Aeróbia, Escola de Educação Física e Esporte, Universidade de São Paulo, São Paulo, SP, Brasil; 2 Grupo de Pesquisa em Desempenho Humano, Universidade Tecnológica Federal do Paraná, Curitiba, PR, Brasil

**Keywords:** Endurance exercise, Oxygen uptake, Muscle response, Tissue oxygen saturation, Peripheral oxygen extraction

## Abstract

The present study investigated the reliability and sensitivity of a wearable near-infrared spectroscopy (wNIRS) device in moderate and heavy exercise intensity domains. On three separate days, eleven males performed an incremental test to exhaustion, and in the following visits, four submaximal constant-load bouts (i.e., test and retest) were performed in the moderate-intensity domain (100 and 130 W) and heavy-intensity domain (160 and 190 W). The local tissue oxygen saturation index (SmO_2_) and pulmonary oxygen uptake (V̇O_2_) were measured continuously. The absolute SmO_2_ and V̇O_2_ values and the change (Δ) from the 3rd to 6th min of exercise were calculated. There was good reliability for SmO_2_ measurements, as indicated by the high intraclass correlation coefficient analysis (ICC ≥0.84 for all) and low coefficient of variation between the two trials (CV ≤4.1% for all). Steady-state responses were observed for SmO_2_ and V̇O_2_ from the 3rd to the 6th min in the two moderate-intensity bouts (P>0.05), whereas SmO_2_ decreased and V̇O_2_ increased from the 3rd to the 6th min in the two heavy-intensity bouts (P<0.05). Together, these findings suggested that the SmO_2_ measured with a wNIRS device is reliable and sensitive to track local metabolic changes provoked by slight increments in exercise intensity.

## Introduction

The number of recreational endurance athletes has increased considerably over the last decades, ultimately fomenting the need for different evaluation methods to monitor the acute and long-term physiological responses to endurance exercise. Laboratory-based methods have been used to measure physiological responses based on pulmonary oxygen uptake (V̇O_2_) responses during constant-load exercises of different intensities (1). It has been widely assumed that the V̇O_2_ response represents a dynamic change in O_2_ delivery and utilization within the working muscles (2). Previous findings showed that the V̇O_2_ response during the moderate-intensity exercise domain (i.e., below the gas exchange threshold, GET) is characterized by a monoexponential increase in V̇O_2_ that usually reaches a steady state within 3 min in healthy subjects (3). If exercise intensity surpasses the metabolic demand associated with the GET but remains below the respiratory compensation point (RCP), the V̇O_2_ steady state is delayed, resulting in a significant increase in V̇O_2_ from the 3rd to 6th min of exercise (2).

However, V̇O_2_ measurement is extremely costly and requires highly skilled technicians to collect and interpret the data, ultimately limiting its broad utilization in simple clinical and sport testing facilities. Among the most promising alternative approaches for tracking muscle metabolic demands during exercise, near-infrared spectroscopy (NIRS) is of utmost interest since it provides noninvasive continuous monitoring of O_2_ saturation in the microvasculature surrounding the active musculature (4,5). Recent evidence has suggested that NIRS is a valid tool for characterizing the dynamics of muscle O_2_ changes (i.e., the balance between O_2_ delivery and extraction by the skeletal muscles) among different exercise intensities (6,7). For example, Belardinelli et al. (8) observed higher muscle O_2_ saturation index (SmO_2_) values in exercise intensities within the moderate-intensity domain compared to the heavy-intensity domain, thus showing that NIRS is sensitive for tracking muscle O_2_ saturation changes with exercise intensity.

Wearable technology devices, such as fitness trackers, smart watches, and heart rate monitors, have become the most important global fitness trends for 2020 (9). These devices are relatively inexpensive and require minimal technological skills from users to analyze and interpret the data (10). They have been recently used to monitor SmO_2_ during different sport scenarios, such as indoor rock climbing (11), cross-country skiing (12), and cycling (5). However, while there are a large number of studies showing the reliability and sensitivity of NIRS (13,14), data showing the reliability and sensitivity of wearable NIRS devices (wNIRS) are lacking (15,16). Before using new devices for physiological analysis, it is crucial to establish their reliability. Reliability is defined as the extent to which measurements can be replicated (17), and it has often been characterized by data consistency between test-retest measurements for wNIRS variables (5). Sensitivity, determined by ability to detect the effects of exercise intensity in the wNIRS variables (5), is another parameter that needs to be established before the use of any assessment tools. To our knowledge, only one study has reported the validity of a wNIRS device (Humon, USA) in monitoring muscle O_2_ dynamic changes during an exercise bout (16). The study design, however, was limited to an incremental test protocol that did not allow us to investigate steady-state responses during different intensity exercise domains (18). From a practical perspective, this is equally important because most active individuals and endurance athletes are not usually trained using incremental workloads. Thus, assessing the reliability and sensitivity of wNIRS to measure SmO_2_ during exercise at moderate- and heavy-intensity domains is of utmost relevance, as determining the effect of a program on endurance performance enhancement is best accomplished when reliable and sensitive tools are used to track training-induced changes (19).

Therefore, the aim of the present study was to investigate the reliability and sensitivity of a wNIRS device to measure SmO_2_ during constant-load exercise bouts in different exercise-intensity domains. We hypothesized that the device: i) would be highly reliable in a test and retest approach, and ii) would be sensitive in detecting SmO_2_ changes across different exercise intensities.

## Material and Methods

### Participants

The sample size was estimated using G*power software (version 3.1.9.2, Germany) from data of a previous study that compared muscle oxygen saturation at different exercise intensities (i.e., at maximal lactate steady state intensity and 15% above that level) (20). A sample size of eight participants was estimated to be sufficient to detect the significant effect of exercise intensity on muscle oxygen saturation for a power of 0.90 and alpha level of 0.05. Thus, eleven young males (n=11) were recruited to participate in this study (age: 23±2 years; height: 176.5±5.9 cm; body mass: 82.4±9.9 kg; body fat: 16±2%; maximal oxygen uptake (V̇O_2max_): 40.5±3.4 mL/kg/min; peak power output: 288.2±35.7 W; vastus lateralis (VL) muscle thickness: 12±5 mm). Considering that light interaction with human tissue can be influenced by individual factors (such as skin color) (21), participants were asked to self-report their skin color. All participants reported having white skin color. The procedures, including the experimental protocol, benefits, and risks, were explained before the participants gave their written informed consent. The study was conducted according to the Declaration of Helsinki and was approved by the Research Ethics Committee (process No. 3235882) of the School of Physical Education and Sport, University of São Paulo, Brazil.

### Experimental design

**Figure 1:**
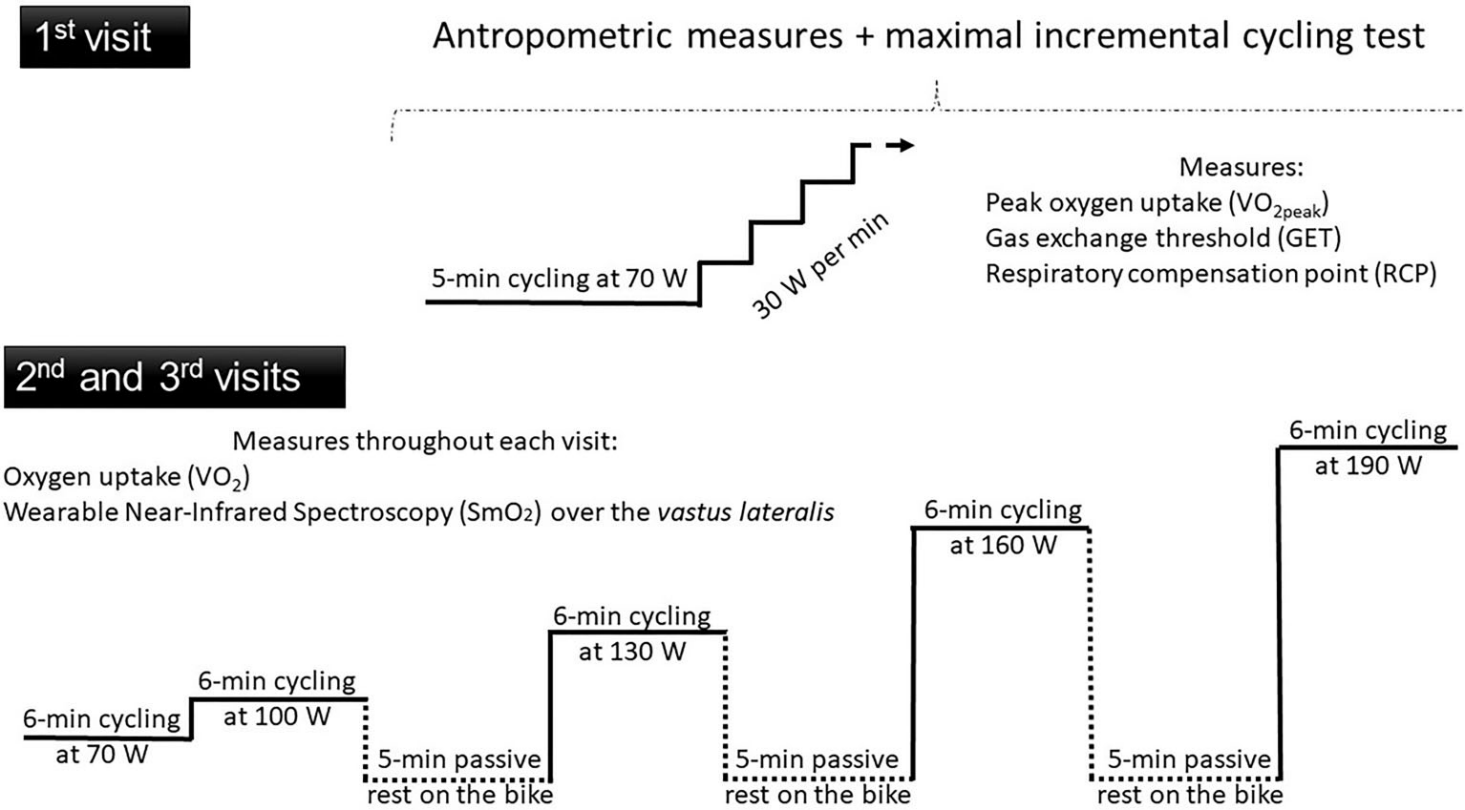
Experimental design overview.

The participants visited the laboratory on three separate occasions (48 h apart) at the same time of day (Figure 1). In the first visit, anthropometric measurements were taken and a maximal incremental cycling test to exhaustion was performed. In the second and third visits (test and retest, respectively), participants performed a 6-min warm-up at 70 W followed by four 6-min bouts of constant-load exercise. The first two bouts were performed at moderate intensity (i.e., below the GET), while the last two bouts were performed at heavy intensity (i.e., between the GET and RCP). Those exercise intensities were chosen to have a wide range of physiological responses around the GET, which is commonly used to prescribe aerobic training interventions and represents the boundary between the moderate and heavy domains (22). Participants were asked to have the last meal 2 h before the tests and refrain from exhaustive exercise, consumption of alcohol, and caffeine intake 48 h before the tests. They also completed a 48-h food diary during the two days before the second visit and were instructed to replicate their recorded diet during the 48 h before the third visit.

### Procedures

#### Anthropometric measurements

Anthropometric measurements were performed according to the procedures suggested by Norton and Olds (23). Body mass and height were measured using an electronic scale and a stadiometer with 0.1 kg and 0.1 cm accuracy, respectively. Skin thickness was measured by a compass (Harpenden, UK) to estimate local VL thickness, and body fat percentage was estimated according to Brozek et al. (24).

#### Maximal incremental cycling test

The tests were carried out with a bicycle attached to a cyclosimulator (RacerMate^®^, Computrainer™, USA). The cyclosimulator was calibrated before each test in accordance with the manufacturer's recommendations. After a 5 min warm-up at 70 W, the power output was increased by 30 W every 1 min until voluntary exhaustion. The pedal cadence was maintained between 80-90 rpm, with voluntary exhaustion being defined as the inability to sustain the minimum cadence of 80 rpm for more than 5 s even with strong verbal encouragement. Gas exchange responses during maximal and submaximal cycling tests were measured breath-by-breath throughout the trials using an automatic metabolic cart (Cortex Metalyzer 3B, Cortex Biophysik, Germany). The metabolic cart was calibrated prior to the tests using a 3-L syringe, ambient air, and a cylinder containing known concentrations of O_2_ and CO_2_ (12 and 5%, respectively).

#### Constant-load tests

The procedures related to cycling, warm-up, and gas exchange responses were similar to those adopted in the maximal incremental cycling test. The moderate- and heavy-intensity domains were identified as workload below the GET value and between the GET and RCP, respectively (25). The participants performed two bouts in the moderate-intensity (i.e., 100 and 130 W) domain and two bouts in the heavy-intensity domain (i.e., 160 and 190 W), with 5-min passive rest between bouts. These intensities corresponded to 35.2±4%, 45.7±5.2%, 56.2±6.5%, and 66.8±7.7% of peak power output measured during the maximal incremental cycling test, respectively. Participants were instructed to maintain a cadence of 70 rpm during exercise. Throughout the constant-load tests, whole body V̇O_2_ and SmO_2_ over the VL muscle were continuously recorded.

#### Wearable Near Infrared Spectroscopy device

The device (Humon Hex, Dynometrics Inc., USA) was used to monitor the SmO_2_ over the VL belly. Humon Hex was recently validated (16). Briefly, this system is composed of two infrared transmitter LEDs and three photodetectors spaced by 1.2, 1.8, and 2.4 cm from the LEDs. The acquisition rate was set to 4 Hz, and the data were exported second by second. This device communicates with smartphones via bluetooth through an app developed by the manufacturer, enabling real-time viewing. The wNIRS probe was placed on the belly of the right-leg VL muscle midway between the greater trochanter and the proximal border of the patella. Before removing the probe on the first visit, a skin-safe pen was used to draw a line around the probe's placement area. To ensure consistent placement of the probe, the procedures of localization between the greater trochanter and the proximal border of the patella were replicated in all experimental sessions. Double-sided tape and an elastic strap were used to fix the wNIRS probe and prevent movement. Additionally, the wNIRS probe was covered with an optically dense black vinyl film to minimize possible penetration of extraneous light.

#### Data analysis

V̇O_2_ data for the maximal and submaximal cycling exercises were individually analyzed as previously described (26). V̇O_2_ data were cleaned by removing aberrant data points that were three standard deviations (SD) from the local mean and linearly interpolated to 1 s intervals. From the incremental test, V̇O_2max_ was defined as the highest V̇O_2_ computed from a 20-s rolling average. The GET and RCP were identified by 3 independent investigators by examining raw respiratory data, as previously suggested (27). To account for the time delay for deoxygenated hemoglobin to reach the lungs from the active musculature, the kinetics of V̇O_2_ (i.e., mean response time, MRT), and the loss of muscle contraction efficiency during the test, the associated power output to reach GET and RCP was left-shifted on an individual basis, as previously described (28). Peak power output was considered for the highest completed stage at the end of the test. For the submaximal constant-load bouts, V̇O_2_ and SmO_2_ were plotted against time (second-by-second) and reported as the average of the last 30 s of baseline and the average from 2.5 to 3 min and from 5.5 to 6 min of each exercise bout. The slow component of V̇O_2_ kinetics and SmO_2_ were reported as the difference (Δ) between the 3rd and 6th min data of each exercise bout.

### Statistical analysis

All data are reported as the means±SD. Data distribution was verified by Shapiro-Wilk's test, with all variables showing a normal distribution. The reliability of V̇O_2_ and SmO_2_ was determined using the coefficient of variation (CV) and intraclass correlation coefficient (ICC) for baseline and 6th min values. Interpretations of poor (<0.50), moderate (between 0.50-0.75), good (between 0.76 and 0.90), and excellent (>0.90) reliability were adopted for ICC (17). The dynamic changes in V̇O_2_ and SmO_2_ were analyzed by a two-way repeated-measures ANOVA with exercise intensity (100, 130, 160, and 190 W) and time (3rd and 6th min) as factors. The same comparison was made with the difference between the 3rd and 6th min of exercise in each condition. The V̇O_2_ associated with GET was compared to the V̇O_2_ at the 6th min of exercise with a one-way repeated-measures ANOVA, with the main factor as V̇O_2_ in each condition (100, 130, 160, 190 W, and GET). When F values were significant, Tukey *post hoc* tests were used to locate differences. The significance level was set at P≤0.05. All statistical analyses were performed with the SPSS statistical package (version 13.0, IBM, USA).

## Results

### Maximal incremental test

The parameters derived from the incremental test were determined after accounting for the MRT (53±36 s). The absolute and relative V̇O_2_max was 3.30±0.36 L/min and 39.0±4.0 mL^.^kg^-1.^min^-1^, respectively. The peak power output was 288.2±35.7 W, while V̇O_2_ and power output associated with GET and RCP were 2.02±0.16 L/min and 145.5±14.8 W, and 2.72±0.31 L/min and 209±21.2 W, respectively.

### Submaximal constant-load tests

#### Sensitivity

**Figure 2:**
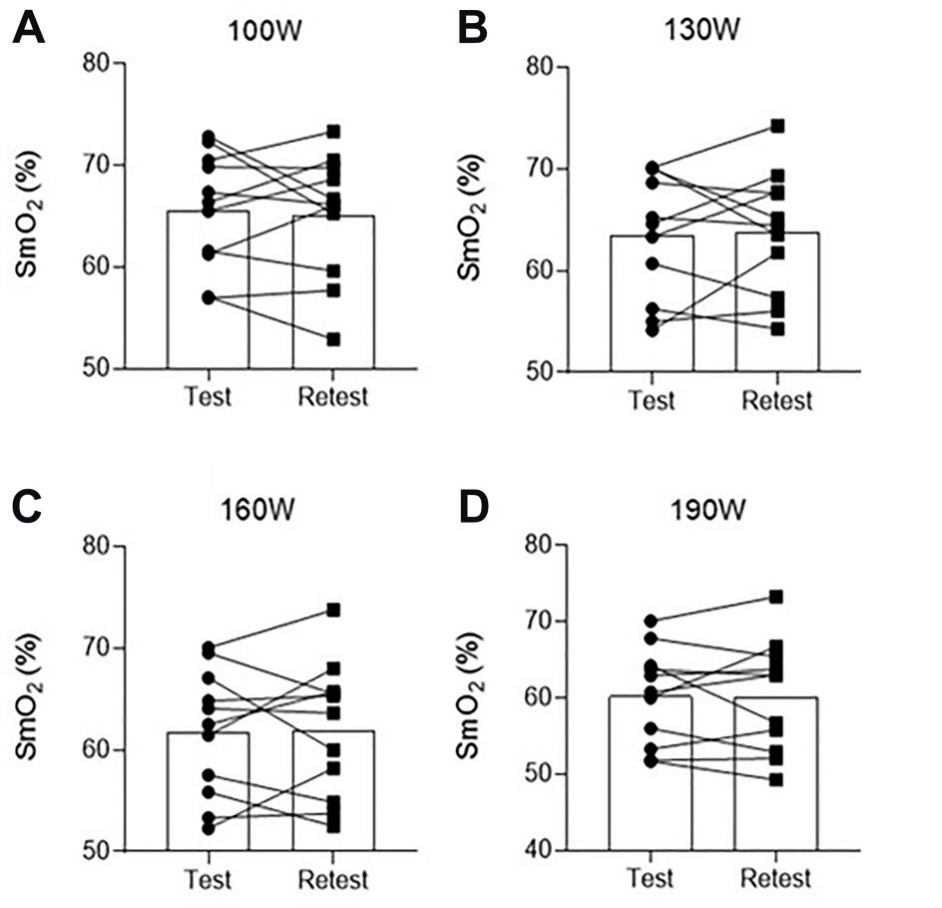
Muscle oxygen saturation (SmO_2_) at the first test and at the retest 48 h after for each exercise bout. Data are reported as mean and individual values.

**Figure 3:**
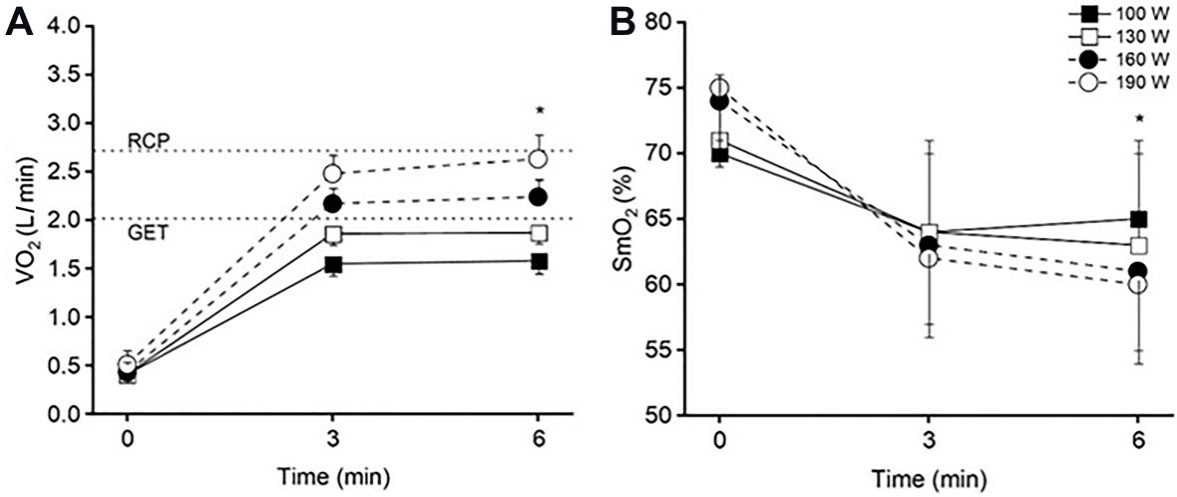
Oxygen uptake (**A**) and muscle oxygen saturation (**B**) at baseline (time zero) and at min 3 and 6 during each exercise bout. Data are reported as means±SD. *P<0.05 from min 3 for 160 and 190 W (ANOVA).

Individual values of SmO_2_ are graphically demonstrated in Figure 2. The dynamic adjustments in V̇O_2_ and SmO_2_ in each exercise bout are shown in Figure 3. There was an interaction effect for V̇O_2_ (P<0.001, $\eta_{P}^{2}$ = 0.55; panel A), in which V̇O_2_ increased from 3 to 6 min at 160 W (P=0.004) and 190 W (P=0.001) but not at 100 W or 130 W (P>0.05). There was also an interaction effect for SmO_2_ (P<0.001, $\eta_{P}^{2}$ = 0.47; panel B), where SmO_2_ from min 3 to min 6 at 160 W (P=0.04) and 190 W (P=0.03), but SmO_2_ was stable at 130 W (P>0.05). Additionally, SmO_2_ increased from 3 to 6 min at 100 W (P=0.02). There was a condition effect for the V̇O_2_ associated with the GET compared to each exercise intensity (P<0.001, $\eta_{P}^{2}$ = 0.91). Whereas the V̇O_2_ at the 6th min of exercise was lower than the GET for 100 W (P<0.001) and 130 W (P=0.04), it was higher than the GET for 160 W (P=0.002) and 190 W (P<0.001) conditions.

There was an interaction effect for ΔV̇O_2_ (P<0.001, $\eta_{P}^{2}$ = 0.56) and ΔSmO_2_ (P<0.001, $\eta_{P}^{2}$ = 0.47). The ΔV̇O_2_ was greater (P<0.05) at 190 W (0.14±0.10 L/min) compared to the other conditions, such as 160 W (0.07±0.06 L/min), 130 W (0.01±0.04 L/min, and 100 W (0.03±0.04 L/min). The ΔSmO_2_ was greater (P<0.05) at 190 W (-1.17±0.68%) than at 130 W (-0.19±0.88%) and 100 W (1.20±1.95%), but not at 160 W (-0.68±0.60%, P=0.31). Additionally, ΔSmO_2_ was positive at 100 W and different from all other intensities (P<0.05).

#### Reliability

Table 1 shows the ICC and CV for SmO_2_ at baseline and 6 min of each exercise intensity. Good-to-excellent values of ICC (0.84-0.96, all P<0.05) and low values of CV (2.0-4.1%) were detected. Table 2 shows the ICC and CV for SmO_2_ at the third minute of each exercise intensity. Good-to-excellent values of ICC (0.77-0.86, all P<0.05) and low CV values (3.0-3.8%) were also detected, revealing the high reproducibility of the wNIRS device for measuring SmO_2._

**Table 1:** Wearable near-infrared spectroscopy reproducibility for SmO_2_ determined at baseline and during two exercise intensities at moderate (100 and 130 W) and heavy (160 and 190 W) domains.

SmO_2_ (%)	Test	Retest	ICC	CV (%)
Baseline	65.2±7.2	65.1±6.6	0.96*	2.0
100 W	65.6±5.7	65.1±6.1	0.86*	3.6
130 W	63.5±6.2	63.8±6.1	0.84*	4.1
160 W	61.7±6.2	61.9±6.7	0.87*	3.9
190 W	60.2±6.3	60.2±7.4	0.91*	3.4

Data are reported as mean±SD of oxygen saturation index (SmO_2_) values on test and retest. ICC: intraclass correlation coefficient; CV: coefficient of variation. *P<0.05.

**Table 2:** Wearable near-infrared spectroscopy reproducibility determined at the third minute of two exercise intensities at moderate (100 and 130 W) and heavy (160 and 190 W) domains.

SmO_2_ (%)	Test	Retest	ICC	CV (%)
100 W	64±6.8	64.1±6.3	0.86*	3.0
130 W	63±5.9	63.5±5.9	0.80*	3.2
160 W	62±6.1	62.8±6.2	0.77*	3.7
190 W	61±6.0	61.4±6.9	0.80*	3.8

Mean±SD of oxygen saturation index (SmO_2_) values on test and retest. ICC: intraclass correlation coefficient; CV: coefficient of variation. *P<0.05.

## Discussion

The main objective of the current study was to investigate the sensitivity and reliability of a wNIRS device in measuring SmO_2_ during constant-load exercise bouts at different exercise intensities. Our main results revealed that: i) the response of SmO_2_ accompanied the V̇O_2_ response among exercise intensities, and ii) there was a high reliability of SmO_2_ in a test-retest format independent of exercise intensity. Taken together, these findings suggested that the device is reliable and sensitive for measuring SmO_2_, independent of exercise intensity.

A main finding of the present study was that SmO_2_ showed a similar time course to the V̇O_2_ response at all exercise intensities. Given that exercise intensity varies considerably throughout endurance events, it is important to understand peripheral O_2_ dynamics. Previous studies have shown that a delayed V̇O_2_ response occurs during exercise performed in the heavy intensity domain (slow component of V̇O_2_), with a steady state being achieved within approximately 6 minutes of exercise (29). A number of putative mechanisms for this delayed V̇O_2_ steady state have been proposed (25,30), including the additional recruitment of type IIb muscle fibers (3,31). Type IIb muscle fibers produce more heat and consume more O_2_ for the same rate of tension generation and ATP resynthesis compared with type Ib muscle fibers (32). This lower energetic efficiency results in a progressive recruitment of type II motor units during heavy exercise, leading to a delayed V̇O_2_ steady state (32).

Our results also revealed that in addition to V̇O_2_, ΔSmO_2_ achieved a steady state at 3 min during moderate intensity exercise but increased from 3 to 6 min during heavy intensity exercise. This was in accordance with Belardinelli et al. (8), who observed a progressive decline in SmO_2_ during heavy-intensity exercise using an NIRS system. Poole et al. (33) reported that the delayed V̇O_2_ steady state during heavy intensity exercise is associated with an increase in blood flow and continued desaturation of femoral venous blood. Collectively, these findings suggest that NIRS-derived SmO_2_ appears to reflect the balance between O_2_ supply in the small vessels and O_2_ consumption by tissues for ATP resynthesis. From a practical perspective, the nonsteady state of NIRS parameters found in the heavy intensity domain might suggest that ΔSI (saturation index) and ΔHHb (deoxy-hemoglobin) determination should be performed with caution in future studies since they are time-dependent and, consequently, different results can be obtained when the timing of data collection and analysis are not standardized. Thus, sports scientists and coaches should be careful to monitor O_2_ extraction by exercised muscles at heavy domain intensities where there is no stabilization of wNIRS parameters.

The increased popularity of endurance events in recent decades has been accompanied by the evolution of equipment used in physiological measurements. However, it is important to determine the reliability of these devices to accurately monitor physiological responses during exercise. A reliable test is one that has a small between-day change in mean values, a small within-individual variation, and a high test-retest correlation (12). The reliability of physiological parameters can be statistically determined in different ways, but the ICC is the most relevant statistical analysis (17). Previous studies analyzing the reliability of other NIRS devices reported similar results to those found in the current study. van der Zwaard et al. (14) demonstrated a high reliability for SmO_2_ measured at different intensities during incremental tests, such as 50% of V̇O_2_max (ICC=0.90, CV=5.2%), 75% of V̇O_2_max (ICC=0.92 and CV=9.1%), and 100% of V̇O_2_max (ICC=0.97 and CV=8.0%). Despite these remarkable findings concerning the reliability of NIRS, at least to our knowledge, there have been no previous studies analyzing whether wNIRS is reliable for measuring SmO_2_ during tests with constant workload. In the current study, we found high reliability for SmO_2_ during both moderate- and heavy-intensity exercise domains. These findings suggested that wNIRS is reliable for measuring SmO_2_ independent of exercise intensity.

Some limitations of the present study must be recognized. First, our participants were physically active men with a low percentage of body fat. This is relevant because the local adipose tissue influences the distance between the light emitter and receptor, affecting hemoglobin and myoglobin monitoring. Second, all participants were white and therefore had little melanin in their skin, which absorbs part of the light in the near-infrared range (21). Considering the small thickness of the thigh (∼11 mm), which is less than that affecting the hemoglobin and myoglobin signal (15 mm) (34), and the presence of little melanin in the skin, the results of the current study should therefore be extrapolated with caution to other populations with higher local adipose tissue and/or greater melanin concentrations in the skin.

In conclusion, SmO_2_ measured by a wNIRS device was reliable and sensitive for detecting time-dependent and intensity-dependent changes in the balance between local O_2_ supply and consumption. From a practical standpoint, these results suggested that the SmO_2_ value derived from wNIRS can be used in clinical and sports performance contexts.
